# No contribution of lifestyle and environmental exposures to gender discrepancy of liver disease severity in chronic hepatitis b infection: Observations from the Haimen City cohort

**DOI:** 10.1371/journal.pone.0175482

**Published:** 2017-04-28

**Authors:** Jing Sun, Lucy Robinson, Nora L. Lee, Seth Welles, Alison A. Evans

**Affiliations:** 1 Department of Epidemiology, Johns Hopkins University Bloomberg School of Public Health, Baltimore, Maryland, United States of America; 2 Department of Epidemiology and Biostatistics, Drexel University Dornsife School of Public Health, Nesbitt Hall, Philadelphia, Pennsylvania, United States of America; Ospedale San Raffaele, ITALY

## Abstract

**Background:**

Previous studies have noted significant gender difference in the risk of liver cancer among hepatitis B chronic infection patients. Some indicated that it might be due to lifestyle-related differences. This paper tests whether or not such a gender discrepancy among the chronic hepatitis B population is confounded by lifestyle and environment related exposures.

**Methods:**

We retrieved a sample of 1863 participants from a prospective cohort in Haimen City, China in 2003. Liver disease severity was categorized as “normal”, “mild”, “moderate”, and “severe” based on a clinical diagnosis. Lifestyle and environmental exposures were measured by questionnaires. We used factor analysis and individual variables to represent lifestyle and environmental exposures. We applied the cumulative logit models to estimate the effect of gender on liver disease severity and how it was impacted by lifestyle and environmental exposures.

**Results:**

Gender and HBeAg positivity were independent risk factors for more severe liver disease. Compared to females, males were 2.08 times as likely to develop more severe liver disease (95% CI: 1.66–2.61). Participants who were HBeAg positivite were 2.19 times (95% CI: 1.61–2.96) as likely to develop more severe liver disease compared to those who were negative. Controlling for lifestyle and environmental exposures did not change these estimations.

**Conclusions:**

Males in the HBV infected population have an increased risk of severe liver disease. This gender effect is independent of the lifestyle and environmental exposures addressed in this study. Our findings support the hypothesis that gender discrepancies in HCC risk are attributable to intrinsic differences between males and females.

## Background

Hepatitis B infection is prevalent worldwide. Over 240 million people have chronic hepatitis B (HBV) infection [1, 2]. HBV chronic infection can lead to increased risk of death from liver cirrhosis or liver cancer [3–6]. Hepatitis B e antigen, which is an indication of HBV viral load replication, has been associated with severe liver disease condition and hepatocellular carcinoma (HCC) [7, 8]. Previous studies have observed that, among hepatitis B chronic infection patients, males are more likely than females to develop and die from HCC [9–11]. In an eight year follow-up cohort study in Haimen City [9], researchers found that males had a 1.8–6.7 fold increased risk of HCC compared to females. Some have speculated that the gender discrepancy may be due to lifestyle-related differences [12], since previous epidemiologic studies have shown that lifestyle-related exposures (e.g. alcohol consumption and smoking habits) increased the risk of hepatocellular carcinoma in hepatitis B infected patients [3, 13].

The purpose of this study is to determine whether lifestyle and environmental related exposures can explain the gender differences in liver disease severity in the chronic hepatitis B (CHB) population. We examined this effect by utilizing cross-sectional data derived from a long-term prospective study in HBV-infected Chinese adults from the Haimen City cohort.

## Methods

### Study population

The data in this study were derived from a prospective cohort study established in 1992–93 in Haimen City, located in the eastern province of Jiangsu, in China [9]. In 2003, the research team invited 2571 surviving HBsAg-positive cohort members for evaluation of current liver disease status. Of these, 1863 (72.5%) participants attended the screening and evaluation. Written consent of the participants was obtained in both 1993 and 2003. The initial cohort study and the 2003 follow-up were reviewed and approved by the Institutional Review Board of the Fox Chase Cancer Center, Philadelphia, PA, USA, the Medical Ethics Review Group of Haimen City, China, and the Ethics Review Committee of the School of Public Health of Fudan University, Shanghai, China. The Drexel University School of Public Health institutional review board approved the secondary data analysis in 2014.

### Measurements

The details of data collection, laboratory examination, and diagnosis criteria were reported in previously published papers based on the same cohort [9, 14]. In brief, trained physicians surveyed all participants on their family history and medical history, collected blood samples for HBV serology (HBsAg, anti-HBs, HBeAg, anti-HBe, anti-HBc) and blood routines, performed physical examinations, and performed abdominal ultrasounds. HBV serology and HBV viral load were assayed on samples collected in 2003. All results were reviewed and extracted by medical professionals. Liver disease severity in this cohort were summarized and categorized as: “normal”, “mild”, “moderate”, “severe”, and “HCC” [14]. Normal was identified as possessing no abnormalities on any test or exam except for HBV markers. Mild was identified as having elevated alanine transaminase (ALT) and/or Alpha-Fetoprotein (AFP) only, but no other abnormal presentation in the physical examination and ultrasound exam. Individuals were classified as moderate if ultrasounds, physical findings or laboratory test results indicated abnormality, but they did not meet the criteria for probable fibrosis/cirrhosis or HCC. Severe was identified as having at least two of the following: 1) Spider nevi, scleral icterus, palmar erythema, ascites, hepatomegaly, or splenomegaly; 2) Thrombocytopenia and/or prolonged (>2 seconds) prothrombin time; 3) Portal vein enlargement (>12 mm) on ultrasound. HCC is identified by the presence of a mass (>2 cm) on ultrasound and AFP >400 ng/ml. For the purpose of the current analysis, we have combined “severe” and “HCC” (total of 17 individuals were diagnosed as HCC in the current sample) as severe liver disease.

Lifestyle and environmental exposure information was collected through interviews in 2003. Smoking habit information included: 1) ever smoked (defined as smoked more than 1 cigarette per day for more than half of a year); 2) age started smoking; 3) number of cigarettes per day. Alcohol consumption information included: 1) current alcohol consumer (defined as drinking three or more times per week over half year); 2) previous alcohol consumer; 3) age started drinking; 4) types of alcohol consumed (alcohol contents over 40% classified as high, alcohol contents between 20%-40% classified as middle, and alcohol contents less than 20% classified as low); 5) alcohol consumption quantity per week (for this analysis, we classified it as ≤2500g per week and >2500g per week). Tea drinking information included: 1) ever frequent tea consumer (identified as drinking tea three or more times per week over half of a year); 2) age started drinking tea; 3) number of cups of tea per week; 4) types of tea drinking (green, black or jasmine). Drinking water information included: 1) current drinking water source; 2) ever drank well water; 3) years drank well water; 4) ever drank river/ditch water; 5) years drank river/ditch water. All lifestyle and environmental exposure variables had less than 2% missing values. Missing values of lifestyle and environmental exposure variables were imputed using single imputation. Missing values of physical examination results were imputed using multiple imputations [15].

### Statistical methods

We used SAS 9.3 for all statistical analyses. All statistical tests were two-sided. We tested the parallel regression assumption [16], and the gender effect on liver disease severity satisfied the assumption. Thus, we applied cumulative logit regression models in the overall analysis. We developed four models using reorientations of lifestyle and environmental exposure variables. In the first model, only age, age quadratic term (age^2^), gender, and HBeAg status were entered into the model. Lifestyle and environmental exposure variables were correlated in our data. In order to reduce the dimension of lifestyle and environmental exposure variables, we applied factor analysis to all lifestyle and environmental exposure variables to create composite variables for model 2. Model 2 consisted of all variables in model 1 and five factors produced in factor analysis (summarized in Tables 1 and 2). In model 3, we first added each individual lifestyle exposure variable (smoking, alcohol consumption, and tea drinking) into Model 1 and calculated the magnitude of change in gender effect on liver disease severity. Since none of the variables changed the magnitude of the gender effect over 5%, we then selected the variables that had the highest magnitude changes related to smoking and alcohol consumption and entered them into model 3. In model 4, we applied the same method to test all lifestyle (smoking, alcohol consumption and tea drinking) and environmental related (drinking water) variables. Again, none of the individual variables showed a change of magnitude on gender effect over 5%. We then selected five binary variables that reflect the previous exposure of smoking, alcohol consumption, tea drinking, and drinking water entered into model 4. Regression coefficients, standard errors, and p-values for each intercept and covariate are reported for each model. Odds ratios (ORs) and 95% Confidence Intervals (95% CIs) for gender, HBeAg, and lifestyle and environmental exposures were reported separately based on additional calculation.

**Table 1:** Individual characteristics and liver disease severity among participants in Haimen City cohort in 2003.

Variable	Total (n = 1863)	Liver Disease Severity				P value
		Normal (n = 1059)	Mild (n = 170)	Moderate (n = 188)	Severe (n = 446)	
**Demographic**						
Age	52.2±17.3	52.9±17.9	50.7±15.6	50.9±16.1	51.8±16.7	<0.01
Gender (%)						<0.01
Male	1051 (56.4)	512 (48.4)	113 (66.5)	127 (67.6)	299 (67.0)	
Female	812 (43.6)	547 (51.7)	57 (33.5)	61 (32.5)	147 (33.0)	
Occupation (%)						0.17
Peasant	1450 (77.8)	830 (78.4)	134 (78.8)	134 (71.3)	352 (78.9)	
Non-peasant	413 (22.2)	229 (21.6)	36 (21.2)	54 (28.7)	94 (21.1)	
**Smoking**						
Ever Smoked (%)						<0.01
Yes	563 (30.2)	270 (25.5)	59 (34.7)	76 (40.4)	158 (35.4)	
No	1300 (69.8)	789 (74.5)	111 (65.3)	112 (59.6)	288 (64.6)	
Smoking start age (N = 563)	22.4±10.4	22.4±10.4	22.9±12.9	23.0±11.5	22.1±8.7	0.91
Cigarette per day (N = 563)	15.3±14.8	15.1±13.8	15.7±14.5	16.0±15.7	15.2±16.1	0.70
**Alcohol**						
Alcohol consumption (%)						<0.01
Never regular consumer	1269 (68.1)	748 (70.6)	122 (71.8)	111 (59.0)	288 (64.6)	
Previous but not current consumer	73 (3.9)	32 (3.0)	6 (3.5)	6 (3.2)	29 (6.5)	
Current consumer	521 (28.0)	279 (26.4)	42 (24.7)	71 (37.8)	129 (28.9)	
Drinking start age (N = 594)	22.7±13.4	23.5±15.7	22.0±11.3	22.5±9.7	21.6±9.6	0.055
Start age≤20 (%)	332 (55.9)	165 (53.1)	29 (60.4)	36 (46.8)	102 (64.6)	<0.03
Start age>20	262 (44.1)	146 (47.0)	19 (39.6)	41 (53.3)	56 (35.4)	
Alcohol spirit (%) (N = 594)						0.35
High	190 (32.0)	91 (29.6)	22 (45.8)	24 (31.2)	53 (34.0)	
Middle	133 (22.4)	70 (22.8)	7 (14.6)	17 (22.1)	39 (25.0)	
Low	265 (44.6)	146 (47.6)	19 (39.6)	36 (46.8)	64 (41.0)	
Quantity per week (×50g) (N = 594)	64.3±113.4	61.3±105.1	52.5±99.8	83.7±146.9	64.5±111.4	0.04
≤2500g per week	314 (52.9)	171 (55.0)	32 (66.7)	34 (44.2)	77 (48.7)	0.05
>2500g per week	280 (47.1)	140 (45.0)	16 (33.3)	43 (55.8)	81 (51.3)	
**Drink tea**						
Regular tea drinker (%)						0.06
Yes	139 (7.5)	68 (6.4)	16 (9.4)	22 (11.7)	33 (7.4)	
No	1724 (92.5)	991 (93.6)	154 (90.6)	166 (88.3)	413 (92.6)	
Age started drinking tea (N = 139)	29.9±23.9	30.7±25.0	28.1±21.0	31.1±25.9	28.2±21.9	0.84
Cups of tea per week	11.1±20.6	10.5±15.0	14.8±38.3	10.6±18.2	11.1±20.5	0.80
Types of tea						0.66
Green	108 (77.7)	48 (70.6)	13 (81.3)	19 (86.4)	28 (84.9)	
Black	24 (17.3)	16 (23.5)	2 (12.5)	3 (13.6)	3 (9.1)	
Jasmine	5 (3.6)	3 (4.4)	1 (6.3)	0	1 (3.0)	
**Drinking water**						
Current drinking water (%)						0.33
Tap	1844 (99.0)	1051 (99.2)	167 (98.2)	185 (98.4)	441 (98.9)	
Well	19 (1.0)	8 (0.8)	3 (1.8)	3 (1.6)	5 (1.1)	
Drank well water before (%)						0.91
Yes	1445 (77.6)	816 (77.1)	133 (78.2)	145 (77.1)	351 (78.7)	
No	418 (22.4)	243 (23.0)	37 (21.8)	43 (22.9)	95 (21.3)	
Years drank well water (N = 1445)	10.2±14.9	9.7±13.1	10.0±13.2	10.6±17.5	11.2±17.9	0.25
Drank river/ditch water before (%)						<0.01
Yes	1826 (98.0)	1047 (98.9)	166 (97.7)	183 (86.2)	430 (96.3)	
No	36 (1.9)	12 (1.1)	3 (1.8)	5 (2.7)	16 (3.6)	
Years drank river/ditch water before (N = 1826)	31.8±21.8	32.6±22.1	29.9±21.1	30.1±21.1	31.2±21.3	<0.01
Family history of liver cancer (%)						0.04
Yes	337 (18.1)	189 (17.9)	27 (15.9)	24 (12.8)	97 (21.8)	
No	1526 (81.9)	870 (82.2)	143 (84.1)	164 (87.2)	349 (78.3)	

Categorical variables were presented as number of subjects (row percentage) and tested by Fisher’s exact test. For numeric variables, the variables with normal distribution were expressed as mean ± SD and tested by independent samples Wilcoxon-Mann-Whitney test.

**Table 2:** Lifestyle and environmental factors explained by loading of variables from factor analysis.

	Factor1	Factor2	Factor3	Factor4	Factor5
**Ever smoker**	**0.62556**	0.09444	-0.12134	**-0.62358**	0.28530
**Cigarettes per day**	**0.60960**	0.05639	-0.10188	**-0.59910**	0.25977
**Smoking start age**	**-0.60371**	-0.01515	0.19765	**0.65794**	-0.18795
**Ever consume alcohol**	**0.87893**	0.36091	0.02742	0.20065	-0.10444
**Drinking start age**	**-0.83303**	-0.26256	0.06921	-0.09145	0.16074
**Age stop consume alcohol**	0.17633	0.10459	-0.07464	-0.10195	0.03582
**Quantity of alcohol consumption per week**	**0.69191**	0.28003	0.05864	0.23118	-0.12105
**Current alcohol consumer**	**0.83365**	0.33041	0.05838	0.23769	-0.11101
**High alcohol spirit consumer**	0.43164	0.10168	-0.03141	-0.16932	0.08249
**Middle alcohol spirit consumer**	0.33733	0.18746	0.01193	0.20865	0.03180
**Low alcohol spirit consumer**	**0.54614**	0.25037	0.05937	0.27246	-0.23754
**Never regular alcohol consumer**	**-0.87840**	-0.35823	-0.03078	-0.21010	0.10719
**Ever tea consumer**	**0.48049**	**-0.81826**	0.19425	0.11943	0.04564
**Age started drink tea**	-0.32648	**0.62074**	0.04980	0.07330	0.15011
**Cups of tea per day**	0.39848	**-0.66219**	0.21023	0.04511	0.06366
**Never regular tea consumer**	**-0.48032**	**0.81876**	-0.19222	-0.12113	-0.04396
**Green tea consumer**	0.43816	**-0.73487**	0.14619	0.12164	0.06739
**Black tea consumer**	0.15834	-0.32102	0.10963	0.01793	-0.03627
**Jesmine tea consumer**	0.09903	-0.11169	0.07048	0.02262	-0.00360
**Ever drank well water**	-0.10330	-0.13612	-0.42731	0.00428	-0.40341
**Current well water consumer**	-0.10175	0.11226	**0.69263**	-0.40869	**-0.56654**
**Year drank well water**	0.00151	-0.21422	**-0.58152**	-0.04508	**-0.50125**
**Ever drank river/ditch water**	-0.11909	0.17276	0.39559	0.07426	0.36506
**Year drank river/ditch water**	-0.06343	0.28672	**0.50281**	0.19890	**0.51422**
**Current river/ditch water consumer**	0.10175	-0.11226	**-0.69263**	0.40869	**0.56654**

The loading coefficient in bold showed in the table indicated the loading is greater than 0.45 or less than -0.45

## Results

### Characteristics of the study population

Table 1 presents the basic characteristics of the participants and their prevalence at each level of liver disease severity. There are 1863 HBV infected participants in this study, with 56.8% classified as normal, 9.1% classified as mild, 10.1% classified as moderate, and 23.9% classified as severe liver disease. The age range of the participants is 34 to 76 with a mean age of 52.2±17.3, and there is significant age difference among liver disease severity groups (P-value<0.01). Among the 1863 participants, 56.4% were male and 43.6% were female. Males were more likely to have severe liver disease. Among those who had normal liver condition, only 48.5% were males, while among those who had mild, moderate and severe liver disease, over 66% were males (p-value<0.01). Males were also more likely to have HBeAg positive. The prevalence of HBeAg positivity was 8.8% within the population and 7.0% among females, while 10.2% among males.

The overall prevalence of smoking was 30.2% (563). The prevalence of ever smoking was higher in the moderate and severe liver disease categories compared to the normal and mild categories (P-value<0.01). Among all participants, 1269 (68.1%) did not drink alcohol regularly, 73 (3.9%) previously had but did not currently drink alcohol, and 521 (28.0%) were current alcohol consumers. The prevalence of past alcohol consumption was higher among those with more severe liver disease (P-value<0.01). Among those who ever drank alcohol, more than half started drinking by age of 20 (55.9%), and this proportion was even higher among those with severe liver disease (64.6%). About 47% of those who drank alcohol reported they consumed over 2500g alcohol per week. There are significant differences in alcohol consumption quantity between liver disease severity groups (P-value for Fisher’s exact test was 0.05). There were 98% of the participants who claimed that they had consumed river or ditch water before. Those who had not drank river/ditch water were more likely to have a severe liver disease condition (P-value for Fisher’s exact test <0.01).

### Relationship between lifestyle and cnvironmental exposures and gender

In this study, multiple lifestyle and environment related exposures showed statistically significant differences by gender (detail showed in S1 Table). These variables included HBV virus activity (HBeAg status and HBV viral load), smoking (had ever smoked, smoking start age, quantity smoked per day), alcohol consumption (drank alcohol previously, drinking start age, quantity of alcohol consumption per week), tea consumption (had ever been frequent tea drinker, age started drinking tea, quantity of tea drinking per week, types of tea), drinking water (drank well water previously, and years drank river/ditch water before). Most had a p-value less than or equal to 0.05 in Fisher’s exact test or Spearman correlation test.

### Factor analysis on lifestyle and environmental exposures

**Fig 1:**
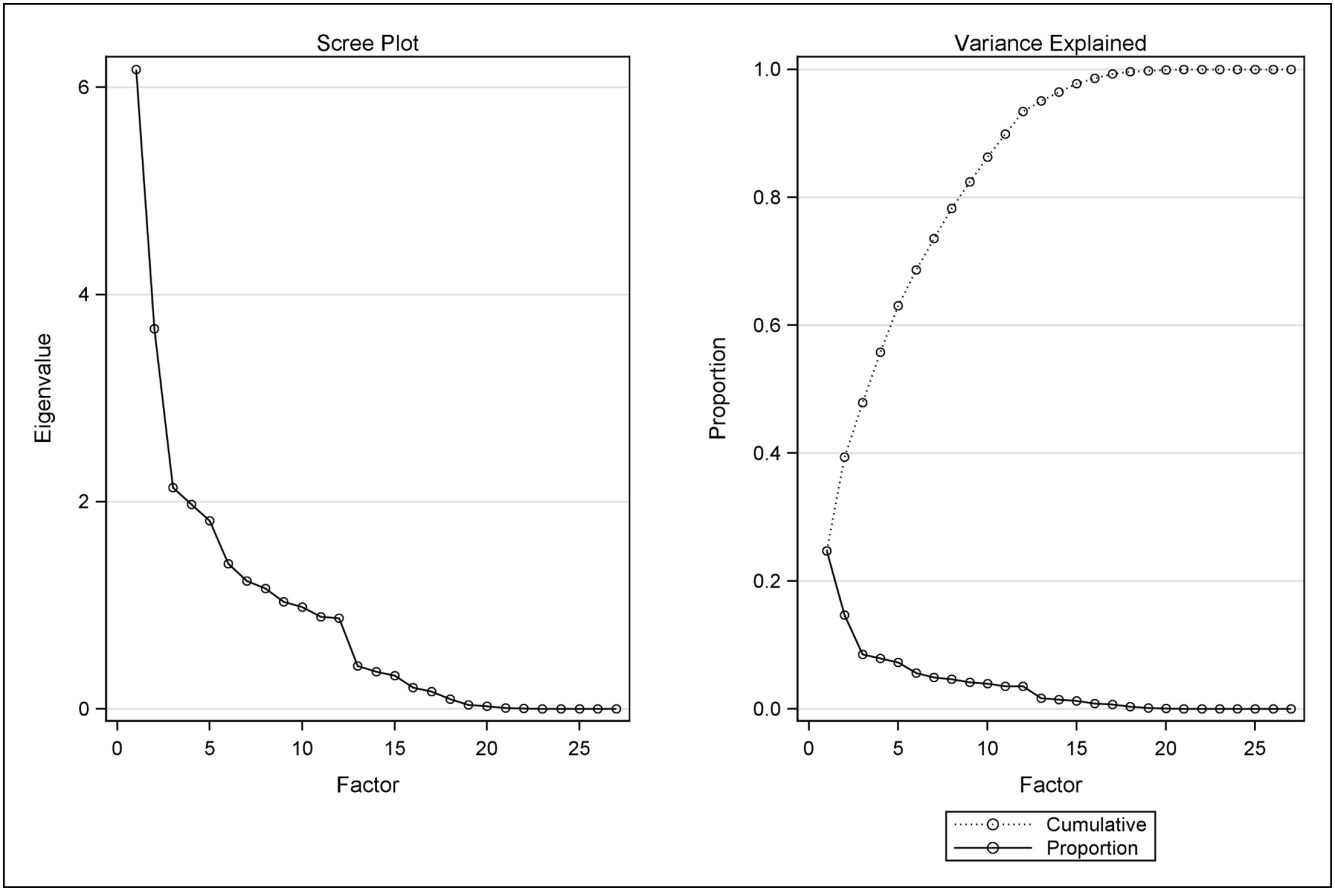
Cumulative and individual proportion of variance explained by each lifestyle and environmental factors in factor analysis. Figure located on the left shows a scree plot that displays the eigenvalues that each factor associated with. Figure located on the right shows proportion of variance each factor and cumulative of all factors explained in the data.

Factor analysis was performed using 28 variables (summarized in Table 2) related to lifestyle and environmental exposure. We generated factors that represent the different dimensions of lifestyle and environmental exposure variables based on the variance covariance matrix of these variables. Fig 1 shows the scree plots for cumulative and individual proportion of variances explained by each factor. We evaluated the scree plots, variances explained by each factor, and the model fit when fitting different combinations of factors in cumulative logit models. We then decided to retain five factors, which explained 63% of the variance of all lifestyle and environmental exposures, from the factor analysis. The results from factor analysis and loading for all variables in each factor are presented in Table 2. Factor 1 (explaining 24.7% of the variance) mostly reflected loading of variables related to smoking and alcohol consumption. Factor 2 (explaining 14.7% of the variance) mostly reflected the loading of variables related to tea drinking. Factor 3 (explaining 8.6% of the variance) mostly reflected the loading of variables related to drinking water. Factor 4 (explaining 7.9% of the variance) mainly reflected the loading of variables related to smoking. Factor 5 (7.3% of the variance) mainly reflected the loading of variables related to drinking water. Although factor 3 and factor 5 both mainly reflect the loading of variables related to drinking water, they represent a different direction of the dimensions of these variables (Table 2).

### Cumulative logit regression models

Table 3 shows results from four cumulative logit regression models. Among four models, model 4 had the lowest Akaike information criterion (AIC) of 4050. Age was an independent risk factor for liver disease severity across four models. Age-squared was included in the models because age is not linearly associated with prevalence of liver disease severity. Gender and HBeAg status are also independent risk factors for more severe liver disease. Male gender is associated with a 2-fold increase (OR: 2.08, 95% CI: 1.65–2.63 based on model 2) of odds of mor severe liver disease compared to women. Participants who were HBeAg positive were 2.19 times (95% CI: 1.61–2.96) as likely to have more severe liver disease compared to those who were negative.

**Table 3:** Cumulative logit regression model of liver disease severity and risk factors.

Variable	Model 1AIC: 4052.8		Model 2AIC: 4058.7		Model 3AIC: 4055.6		Model 4AIC: 4050.9	
	β (SE)	P value	β (SE)	P value	β (SE)	P value	β (SE)	P value
Intercept (severe)	-4.78 (1.55)	<0.01	-4.96 (1.56)	<0.01	-4.64 (1.59)	<0.01	-3.93 (1.67)	0.02
Intercept (moderate)	-4.27 (1.55)	<0.01	-4.45 (1.56)	<0.01	-4.12 (1.59)	<0.01	-3.42 (1.67)	0.04
Intercept (mild)	-3.86 (1.54)	0.01	-4.04 (1.56)	<0.01	-3.72 (1.59)	0.02	-3.01 (1.67)	0.07
Age	0.14 (0.06)	0.02	0.15 (0.06)	0.01	0.14 (0.06)	0.01	0.15 (0.06)	0.01
Age*Age	-0.0015 (0.0005)	<0.01	-0.0015 (0.0005)	<0.01	-0.0015 (0.0005)	<0.01	-0.002(0.0005)	<0.01
Gender								
Female	0		0		0		0	<0.01
Male	0.74 (0.1)	<0.01	0.72 (0.11)	<0.01	0.72 (0.12)	<0.01	0.76 (0.12)	
HBeAg								
Positive	0.80 (0.15)	<0.01	0.79 (0.16)	<0.01	0.78 (0.16)	<0.01	0.78 (0.16)	<0.01
Negative	0		0		0		0	
Factor 1			0.01 (0.05)	0.84				
Factor 2			0.001 (0.04)	0.98				
Factor 3			-0.04 (0.05)	0.35				
Factor 4			0.08 (0.04)	0.09				
Factor 5			0.011 (0.05)	0.80				
**Smoking**								
Ever Smoked								
Yes					-0.0001 (0.2)	0.99	0.06 (0.12)	0.63
No					0		0	
Cigarette per day (N = 563)					0.005 (0.01)	0.65		
**Alcohol**								
Never regular consumer					0		0	
Previous but not current consumer					0.41 (0.25)	0.11	0.47 (0.23)	0.04
Current consumer					-0.18 (0.15)	0.22	-0.09 (0.11)	0.43
Drinking start age								
Start age≤20					0	0.40		
Start age>20					-0.14 (0.16)			
**Drank Tea**								
Yes							-0.02 (0.17)	0.91
No							0	
**Ever drank well water**								
Yes							0.15 (0.11)	0.19
No							0	
**Ever drank river/ditch water**								
Yes							-0.73 (0.31)	0.02
No							0	

SE: standard error

Model 1: included age age*age gender HBeAg

Model 2: included all variables from Model 1 and factor1-5 from factor analysis

Model 3: included all variables from Model 1 and individual variables (ever smoked, cigarette per day, ever drank alcohol, age started drinking alcohol)

Model 4: included all variables from Model 1 and individual variables (ever smoked, ever drank alcohol, ever drank tea, ever drank well water, and ever drank river/ditch water

Among all lifestyle and environmental exposures examined, previous but not current regular alcohol consumption was an independent risk factor for more severe liver disease in model 4. Compared to women who never drank alcohol, male who previously drank alcohol and currently drank alcohol associated with 5.33 (95% CI: 2.11–13.46) and 8.54 (95% CI: 2.16–33.73) times increased odds of having more severe liver disease, respectively (Table 4). Compared to women who never smoke, men who have smoked before were associated with 2.20 (95% CI: 1.73–2.81) times increased odds of having more severe liver disease (Table 4). Surprisingly, drinking river/ditch water appeared to be a protective factor in model 4. We did not find drinking well water to be associated with liver disease severity in model 4 (P-value = 0.19). Overall, the magnitude of the effect of gender on liver disease severity did not change over 5% after adjusting for lifestyle and environmental exposures in models 2–4 compared to model 1 (Table 3). In order to explore if controlling for the HBV viral load group will change the role of lifestyle factors in the association between gender and liver disease severity, we tested two additional models (S2 Table) adjusting for HBV viral load in three categories (undetectable or <1.6×10^3^ copies/mL, low viral load or 1.6×10^3^–10^5^ copies/mL, and high viral load or ≥10^5^ copies/mL). The results of the two additional models agree with the results in the models that did not control for HBV viral load (Table 3).

**Table 4:** Odds ratios for liver disease severity in risk factors by gender.

Risk factors	Female	Male
**HBV viral activity status**		
HBeAg-	References	2.08 (1.66–2.61)
HBeAg+	2.19 (1.61–2.96)	4.54 (3.12–6.61)
**Ever Smoked**		
No	References	2.08 (1.66–2.61)
Yes	1.06 (0.84–1.34)	2.20 (1.73–2.81)
**Alcohol**		
Never regular consumer	References	2.08 (1.66–2.61)
Previous but not current consumer	2.57 (1.03–6.39)	5.33 (2.11–13.46)
Current consumer	4.11 (1.05–16.16)	8.54 (2.16–33.73)
**Drank Tea**		
No	References	2.08 (1.66–2.61)
Yes	0.98 (0.70–1.38)	2.04 (1.38–3.01)
**Ever drank well water**		
No	References	2.08 (1.66–2.61)
Yes	1.16 (0.93–1.44)	2.40 (1.75–3.31)
**Ever drank river/ditch water**		
No	References	2.08 (1.66–2.61)
Yes	0.48 (0.27–0.88)	1.00 (0.53–1.90)

Odds ratios reported in the table used female non-exposure (HBeAg-, nonsmoker, never alcohol drinker, not regular tea drinker, never well water drinker, never river/ditch water drinker) groups as references.

## Discussion

As expected, HBeAg positivity and male gender are independent risk factors for higher prevalence of severe liver disease. Male gender and HBeAg positivity were both associated with about a 2 times greater risk of developing more severe liver disease. Compared to women who had HBeAg negative status, men who had HBeAg positive status were associated with a 4.5 times increase of odds of more severe liver disease condition. The effect of gender on liver disease severity only changed slightly after controlling for lifestyle and environmental exposures, suggesting that the major explanation for the gender discrepancy in liver disease severity is due to endogenous exposures (e.g. hormonal differences) or genetic differences rather than lifestyle or environmental exposures.

Most lifestyle and environmental exposures appeared to correlate with liver disease severity (Table 1) and with gender (S1 Table) in our study. Males were more likely to smoke in our study (52.5% of males have ever smoked and 1.35% of females have ever smoked, P-value<0.01). Current or previous smoking was associated with increased risk of severe liver disease in this population, which is consistent with previous studies [17–19]. The potential mechanism that causes smokers to develop HCC might be due to the multiple mutagenic and carcinogenic components contained in tobacco. Also, cigarettes can cause oxidative stress due to the generation of cytochrome P450E1-associated reactive oxygen species and depletion of endogenous antioxidants [20].

Alcohol consumption was also an independent risk factor for liver disease severity in our study. In our population, females were less likely to have ever been a frequent alcohol consumer (89.2% of females vs. 51.9% of males had never been regular consumers in their life, P-value<0.01). Our results are consistent with previous studies that show excessive alcohol consumption can produce hepatic steatosis, alcoholic hepatitis, and alcoholic cirrhosis [21, 22]. In a study done by Ohishi et. al, alcohol consumption was associated with an approximately 4 times increased risk of HCC after adjusting for hepatitis B or hepatitis C infection [13]. Excess alcohol consumption will induce acetaldehyde formation, which disrupts liver microtubules and increase the liver NADH/NAD+ ratio. This change can induce liver cells to develop fatty degeneration, thus causing hepatic steatosis [22].

We detected an inverse association between drinking river/ditch water and liver disease severity in our population. Previous studies indicated that ditch, pond and river water in the Haimen city region contains certain amount of microcystins, a hepatotoxic peptide produced by algae [23, 24]. Epidemiological studies on the contribution of such compounds in river/ditch water to the high prevalence of HCC in the region have had conflicting results [25, 26]. The inverse association observed in our study might be due to unmeasured confounding factors that related to socio-economic status in the region. At this point, there is no sufficient data to examine this hypothesis, and further studies are needed to validate this potential association. Even though the effect of drinking river/ditch water on liver disease severity was statistically significant based on our analysis, we did not observe that controlling for such effect changed the magnitude of the effect of gender on liver disease severity.

Previous research has suggested an antioxidant component of Chinese green tea that might limit the damage caused by oxygen radicals and inhibit the development of HCC in animal studies [27, 28]. However, the epidemiological studies have reported inconclusive results [28, 29]. Based on our data, we did not observe an association between tea consumption and liver disease severity. The prevalence of frequent tea drinkers was low in our population. Thus we might not have sufficient power to detect such effects. We also did not observe that controlling for tea drinking would change the magnitude of the effect of gender on liver disease.

The gender effect was not eliminated or modified by adjusting for individual or multiple lifestyle and environmental exposures, regardless of the association between lifestyle and environmental exposures and liver disease, and the association between lifestyle and environmental exposures and gender in our population. Based on the results from our multiple models analysis, our study does not support the hypothesis that lifestyle and environmental exposures are the key factors that drive the gender discrepancy in liver disease severity in the CHB population.

Other researchers have indicated that sex-specific hormones might contribute to the gender differences of HCC mortality or morbidity. This hypothesis has been examined in several animal studies [30, 31]. Estrogen exposure has been associated with decreased risk of HCC, while the administration of testosterone accelerated HCC development in an animal study [31]. Pok et al. observed that testosterone enhanced the expression of liver cell cycle regulators, while estradiol suppressed the expression of liver cell cycle markers. Both estrogen and testosterone hormones might jointly determine the gender discrepancy of HCC in mice [31]. On the other hand, work by Kemp et al. indicated that testicular feminized mutant mice, which lack functional androgen receptors, have a similar risk of drug induced HCC compared to female mice but much less than other male mice [32]. In his study, testicular feminized mutant mice developed an average 0.7 liver tumors per animal, while normal males averaged 20 liver tumors per animal. Yu and Chen’s epidemiological study conducted in Taiwan observed that an elevated testosterone level in study participants was associated with an increased risk of HCC [33]. Future epidemiological studies are needed to evaluate the contribution of sex hormones on the association between gender and liver disease severity in the CHB population.

There were some limitations in our study. First, although the subjects were recruited from a prospective cohort study, lifestyle and environmental exposures were measured cross-sectionally at the time of the liver disease examination. Second, the measurements of lifestyle and environmental-related exposures were limited by the questionnaire used. We might miss other potential lifestyle and environmental related exposures that could be unmeasured confounders. However, we have tried several statistical approaches to represent the different features of these variables, and it is unlikely that the estimation of lifestyle and environmental exposures’ impact on the association between gender and liver disease severity would change even if there were more measurements available. In addition, previous studies suggested certain chemical compounds—such as aflatoxin—have hepatotoxic effects which might interact with sex hormones in the human body and might be a potential confounder in this relationship [34]. Future studies should consider incorporating the measurement of serum aflatoxin levels, and the ways it might interact with gender and impact on liver disease severity in the CHB population. Third, we were unable to determine if any changes in lifestyle behavior were attributable to liver disease progression. Previous research has indicated that people with chronic liver damage often experience changes in their sense of taste or smell [35], thus cross sectional estimations might underestimate the effects of smoking and alcohol. Future studies on lifestyle and environmental exposures-related associations in a cohort study design are needed to verify our findings.

There are certain strengths of our paper. This is the first study to use several methods to evaluate the contribution of environmental and lifestyle impacts on the association between gender and liver disease severity in the CHB population based on large cohort data. Our methods provide insight for other similar studies, demonstrating how to summarize complex environmental and lifestyle exposure confounders. Our study provided evidence to support the hypothesis that the gender effect on liver disease severity among CHB is not altered by the behavioral difference between males and females in a large Chinese population. These findings validated the observation in animal studies and other human populations, and it brings the focus of research on gender discrepancy in liver disease severity back to biological pathways. Our findings also suggested alcohol and smoking associated with more severe liver disease condition in a CHB population. Thus, limiting smoking and alcohol consumption would be beneficial for CHB patients’ long term management. Because HBeAg positive males were associated with a greater than fourfold increased odds of severe liver disease, antiviral treatment should be considered at an earlier date than for comparable female CHB patients. Further studies and clinical trials should be performed to evaluate the long term benefit of gender specific management of CHB.

## Conclusion

Based on our observations, male gender increases the risk of liver disease severity in the CHB population. Our results showed that this gender effect is independent of lifestyle and environmental exposures using either factor analysis or controlling for individual variables in a cumulative logit model.

## Supporting information

S1 Table The relationship between HBV virus activity, lifestyle and environmental factors and gender. (DOCX)

S2 Table Cumulative logit regression model of liver disease severity and lifestyle factors, HBeAg status, and HBV viral load. (DOCX)
