# Supplementary material for: No contribution of lifestyle and environmental exposures to gender discrepancy of liver disease severity in chronic hepatitis b infection: Observations from the Haimen City cohort
Source: PLoS One. 2017 Apr 28;12(4):e0175482. doi: 10.1371/journal.pone.0175482 (PMC5409078; doi:10.1371/journal.pone.0175482)
Supplement: S1 Table — (DOCX) [file pone.0175482.s001.docx]

**S1 Table.** The Relationship between HBV virus activity, Lifestyle and Environmental Factors and Gender.

| Variable |  | Gender | | P value |
| --- | --- | --- | --- | --- |
|  | Overall | Male  N=1051 | Female  N=812 |  |
| **HBeAg status** |  |  |  | 0.02 |
| Negative | 1699 (91.2) | 944 (89.8) | 755 (93.0) |  |
| Positive | 164 (8.8) | 107 (10.2) | 57 (7.0) |  |
| **HBV viral load groups** |  |  |  | <0.01 |
| Undetectable (<1.6×103 copies/mL) | 351 (18.8) | 229 (21.8) | 229 (65.2) |  |
| Low viral load(<105 copies/mL) | 925 (49.7) | 423 (45.7) | 502 (47.8) |  |
| High viral load (≥105 copies/mL) | 587 (31.5) | 267 (45.5) | 320 (30.5) |  |
| **Smoking** |  |  |  |  |
| Ever Smoked (%) |  |  |  | <0.01 |
| Yes | 563 (30.2) | 552 (52.5) | 11 (1.4) |  |
| No | 1300 (69.8) | 499 (47.5) | 801 (98.7) |  |
| Smoking start age (N=563) | 22.4±10.4 | 22.2±4.9 | 33.4±12.3 | <0.01 |
| Cigarette per day (N=563) | 15.3±14.8 | 15.4±7.5 | 10.4±5.8 | 0.02 |
| **Alcohol** |  |  |  |  |
| Alcohol consumer (%) |  |  |  | <0.01 |
| Never regular consumer | 1269 (68.1) | 545 (51.9) | 724 (89.2) |  |
| Previous but not current consumer | 73 (3.9) | 56 (5.3) | 17 (2.1) |  |
| Current consumer | 521 (28.0) | 450 (42.8) | 71 (8.7) |  |
| Drinking start age (N=594) |  |  |  |  |
| Start age≤20 (%) | 332 (55.9) | 306 (60.5) | 26 (29.6) | <0.01 |
| Start age>20 | 262 (44.1) | 200 (39.5) | 62 (70.5) |  |
| Alcohol spirit (%) (N=594) |  |  |  | 0.24 |
| High | 190 (32.0) | 164 (32.8) | 26 (29.6) |  |
| Middle | 133 (22.4) | 107 (21.4) | 26 (29.6) |  |
| Low | 265 (44.6) | 229 (45.8) | 36 (40.9) |  |
| Quantity per week (*50g) (N=594) |  |  |  |  |
| ≤2500g per week | 314 (52.9) | 239 (47.2) | 75 (85.2) | <0.01 |
| >2500g per week | 280 (47.1) | 267 (52.8) | 13 (14.8) |  |
| **Drink tea** |  |  |  |  |
| Drinking tea (%) |  |  |  | <0.01 |
| Yes | 139 (7.5) | 126 (12.0) | 13 (1.6) |  |
| No | 1724 (92.5) | 925 (88.0) | 799 (98.4) |  |
| Age started drinking tea (N=139) | 29.9±23.9 | 29.6±12.2 | 32.5±12.6 | 0.31 |
| Cups of tea per week | 11.1±20.6 | 11.5±10.9 | 7.1±2.5 |  |
|  |  |  |  | 0.15 |
| Types of tea |  |  |  | 0.30 |
| Green | 108 (77.7) | 71.9 (79.4) | 8 (61.5) |  |
| Black | 24 (17.3) | 20 (15.9) | 4 (30.8) |  |
| Jasmine | 5 (3.6) | 4 (3.2) | 1 (7.7) |  |
| **Drinking water** |  |  |  |  |
| Current drinking water (%) |  |  |  | 0.06 |
| tap | 1844 (99.0) | 1036 (98.6) | 808 (99.5) |  |
| well | 19 (1.0) | 15 (1.4) | 4 (.5) |  |
| Drank well water before (%) |  |  |  | <0.01 |
| Yes | 1445 (77.6) | 786 (74.8) | 659 (81.2) |  |
| No | 418 (22.4) | 265 (25.2) | 153 (18.8) |  |
| Years drank well water (N=1445) | 10.2±14.9 | 10.5±8.7 | 9.8±6.1 | 0.83 |
| Drank river/ditch water before (%) |  |  |  | 0.15 |
| Yes | 1826 (98.0) | 1025 (97.5) | 801 (98.7) |  |
| No | 36 (1.9) | 25 (2.4) | 11 (1.4) |  |
| Years drank river/ditch water before (N=1826) | 31.8±21.8 | 32.4±11.3 | 31.0±10.9 | <0.01 |

Categorical variables were presented as number of subjects (row percentage) and tested by Fisher’s exact test. For numeric variables, the variables with normal distribution were expressed as mean ± SD and tested by independent samples Wilcoxon-Mann-Whitney test.
