# Supplementary material for: No contribution of lifestyle and environmental exposures to gender discrepancy of liver disease severity in chronic hepatitis b infection: Observations from the Haimen City cohort
Source: PLoS One. 2017 Apr 28;12(4):e0175482. doi: 10.1371/journal.pone.0175482 (PMC5409078; doi:10.1371/journal.pone.0175482)
Supplement: S2 Table — (DOCX) [file pone.0175482.s002.docx]

**S2 Table.** Cumulative logit regression model of liver disease severity and lifestyle factors, HBeAg status, and HBV viral load

| Variable | Model 1  AIC: 4153.97 | | Model 2  AIC: 4153.97 | |
| --- | --- | --- | --- | --- |
|  | β (SE) | P value | β (SE) | P value |
| Intercept (severe) | -4.49 (1.56) | <0.01 | -5.12 (1.58) | <0.01 |
| Intercept (moderate) | -3.97 (1.56) | 0.01 | -4.60 (1.58) | <0.01 |
| Intercept (mild) | -3.55 (1.56) | 0.02 | -4.18 (1.58) | <0.01 |
| Age | 0.12 (0.06) | 0.048 | 0.13 (0.06) | 0.03 |
| Age*Age | -0.0013 (0.0005) | 0.02 | -0.0013 (0.0005) | 0.01 |
| Gender |  |  |  |  |
| Female | 0 |  | 0 |  |
| Male | 0.78 (0.1) | <0.01 | 0.76 (0.12) | <0.01 |
| HBeAg |  |  |  |  |
| Positive | 0.30 (0.17) | 0.08 | 0.29 (0.17) | <0.09 |
| Negative | 0 |  | 0 |  |
| HBV viral load undetectable (<1.6×10^3^ copies/mL) | 0 |  | 0 |  |
| Low HBV viral load(1.6×10^3^ -10^5^ copies/mL) | 0.03 (0.82) | 0.82 | 0.06 (0.13) | 0.66 |
| High HBV viral load (≥10^5^ copies/mL) | 0.76 (0.14) | <0.01 | 0.79 (0.14) | <0.01 |
| Factor 1 |  |  | 0.02 (0.05) | 0.64 |
| Factor 2 |  |  | -0.046 (0.05) | 0.32 |
| Factor 3 |  |  | -0.14 (0.05) | <0.01 |
| Factor 4 |  |  | -0.041 (0.05) | 0.39 |
| Factor 5 |  |  | -0.10 (0.05) | 0.04 |

SE: standard error

Model 1: included age age*age gender HBeAg and HBV viral load

Model 2: included all variables from Model 1 and factor1-5 from factor analysis
